# Year-to-year variation in organic fertilization effects on soil carbon stabilization and microbial networks

**DOI:** 10.3389/fmicb.2025.1707995

**Published:** 2025-11-12

**Authors:** Mingkun Ma, Hao Yang, Jigang Yang, Shanghong Chen, Fang Lei, Dinghui Liu, Zepeng Yang, Honglin Chen

**Affiliations:** 1 Institute of Agricultural Resources and Environment, Sichuan Academy of Agricultural Sciences, Chengdu, China; 2 Key Laboratory of Agricultural Environment in Southwest Mountain Areas of Ministry of Agriculture and Rural Affairs, Chengdu, China

**Keywords:** organic fertilization, soil organic carbon, labile carbon fractions, stable carbon pools, soil enzyme activities, microbial community structure

## Abstract

**Objective:**

Soil organic carbon (SOC) stabilization is a key process linking soil fertility and climate change mitigation; however, its microbial regulatory mechanisms under organic fertilization remain unclear. This study aimed to elucidate how different rates and combinations of organic fertilization regulate SOC fractions, enzyme activities, and microbial communities in newly reclaimed farmland.

**Methods:**

A two-year field experiment (2024–2025) was conducted under a maize–oilseed rape rotation system in Sichuan, China. Treatments included conventional fertilization, varying rates of organic fertilization, and combined organic–inorganic fertilization. SOC fractions, enzyme activities, and microbial community composition were analyzed, and structural equation modeling was applied to explore the microbial and enzymatic pathways driving SOC stabilization.

**Results:**

Organic fertilization enhanced both labile and stable carbon pools. Readily oxidizable carbon (ROC) and dissolved organic carbon (DOC) increased under moderate inputs, while mineral-associated organic carbon (MAOC) accumulated under higher inputs. Enzyme activities mirrored these changes, with laccase activity enhanced under high organic inputs and cellobiohydrolase suppressed by sole organics but restored under combined fertilization. Microbial analysis showed enrichment of *Proteobacteria*, increased diversity, and year-specific shifts in *Actinobacteriota*. Moderate labile carbon inputs promoted stable carbon formation in the first year, whereas excessive inputs in the second year reduced microbial efficiency, increased network complexity, and weakened stabilization.

**Conclusion:**

Moderate organic fertilization improved rapeseed yield and carbon sequestration efficiency by promoting stable carbon accumulation, oxidative enzyme activity, and functional microbial diversity. These findings reveal the microbial and enzymatic mechanisms underlying SOC stabilization in newly reclaimed farmland and provide practical guidance for balanced fertilization strategies to enhance carbon storage while sustaining crop productivity.

## Introduction

Soil organic carbon (SOC) represents the largest and most active carbon reservoir in terrestrial ecosystems, playing a crucial role in regulating atmospheric CO2 and maintaining climate stability (Lal, 2004; Wu et al., 2024). Its storage capacity is approximately 2,000 Pg (Janzen, 2004), exceeding the combined total of the atmospheric and biotic carbon pools and ranking second only to the oceanic carbon pool. SOC is not only a crucial component of soil fertility and structure, preventing degradation, desertification, erosion, and nutrient leaching, but also exerts a decisive influence on the global carbon balance and climate change due to its vast storage capacity and long-term sequestration potential (Jansson and Hofmockel, 2020).

Organic fertilizers, as green and environmentally friendly fertilizers, have been widely applied. They not only enhance soil fertility but also promote the accumulation and sequestration of SOC (Li et al., 2021b). The nutrients and organic matrix they provide simultaneously create conditions for microbial activity and carbon fraction transformation, thereby driving the expansion and stabilization of soil carbon pools (Li et al., 2021b; Pan et al., 2009). Research has shown that organic amendments enhance active carbon pools, including readily oxidizable carbon (ROC) and dissolved organic carbon (DOC). These pools provide essential substrates for microbial metabolism and serve as precursors for the formation of more stable carbon fractions (Fan et al., 2024). But these effects do not follow a straight pattern. Moderate organic inputs usually help stable fractions increase, while excessive inputs can lower SOC stability by accelerating mineralization or exceeding the soil’s ability to hold carbon (Duan et al., 2023; Kuzyakov, 2010). His pattern shows a dose-dependent response but also reveals two opposite functions of labile carbon: at moderate levels, it promotes the formation of stable organic carbon, whereas at excessive levels, it stimulates microbial activity and enhances SOC decomposition.

The long-term stability of soil organic carbon (SOC) mainly depends on how active carbon pools change into stable carbon fractions (Cotrufo et al., 2013). The stable carbon fractions include mineral-associated organic carbon (MAOC), and heavy fraction organic carbon (HFOC). These fractions together form the stable carbon pool in soils. This pool is important for keeping crops productive and for helping control the climate. But the soil can store only a limited amount of organic carbon. The limit comes from its physicochemical traits, often measured by the carbon saturation deficit (Six et al., 2002; Stewart et al., 2008). Recent studies indicate that the formation of new MAOC is driven more by soil carbon saturation than by the amount of organic inputs (King and Sokol, 2025; Ling et al., 2025). This understanding is consistent with the Microbial Efficiency-Matrix Stabilization (MEMS) framework proposed by Cotrufo et al. (2013), which suggests that only a portion of readily decomposable organic matter can be stabilized as organic carbon through microbial metabolism and mineral binding. This proportion is affected by soil matrix traits and by how many binding sites exist on mineral surfaces. Field studies and combined analyses show that soils with high clay content and rich iron oxides favor MAOC formation. But sandy soils have much lower carbon sequestration efficiency because they lack enough mineral protection (Castellano et al., 2015; Kleber et al., 2015). Furthermore, long-term field studies also show that when soils get close to saturation, extra organic inputs often increase ROC or DOC but do not clearly raise MAOC stocks. Such inputs may even accelerate decomposition of existing SOC through priming effects (Kuzyakov, 2010). These findings indicate that SOC sequestration processes are not solely dependent on increased inputs but are simultaneously constrained by soil saturation levels and physicochemical properties.

Biological and enzymatic processes are the core mechanisms driving SOC transformation and stabilization (Zhang F. et al., 2024). Key enzymes, including cellulase (CBH), laccase (LAC), and polyphenol oxidase (PPO), play central roles in regulating plant residue decomposition and the turnover of soil carbon components (Janusz et al., 2020; Sinsabaugh, 2010; Veres et al., 2015). Organic inputs enhance oxidase activity, accelerating the decomposition of lignin-rich and aromatic compounds and facilitating their incorporation into mineral-associated carbon pools through mineralization. At the same time, organic fertilization reshapes soil microbial communities, with different groups showing distinct ecological preferences under varying carbon source conditions (Fierer et al., 2007; Guo et al., 2025; Tian et al., 2022). For instance, copiotrophs thrive in carbon-rich environments, whereas *Actinobacteria* and *Acidobacteria* are better adapted to long-term or nutrient-limited conditions and contribute significantly to the decomposition of complex organic matter (Bao et al., 2021; Dai et al., 2018). Importantly, recent studies indicate that organic inputs primarily enhance functional diversity rather than simply increasing taxonomic abundance, thereby promoting the assembly of specialized decomposer communities capable of efficiently utilizing complex organic substrates (Francioli et al., 2016; Shu et al., 2022; Whalen et al., 2024).

Changes in microbial community structure and enzymatic processes ultimately influence carbon fixation efficiency and crop yield performance, making them of significant importance at the agronomic level. Therefore, the application of organic fertilizers not only enhances crop yields but also substantially determines the efficiency of soil carbon sequestration (Ndung’u et al., 2021; Xu et al., 2025). However, this effect exhibits pronounced dose dependency: moderate application typically achieves the highest carbon sequestration efficiency, promoting stable organic carbon accumulation while maintaining crop productivity. Conversely, excessive application may increase total soil carbon content but demonstrates diminishing returns due to efficiency declines (Jiang et al., 2018; Xu et al., 2025; Zhang et al., 2025). This indicates that balancing productivity and carbon sequestration is essential in practice to develop rational fertilization strategies.

Newly reclaimed farmland represents an early stage of soil ecosystem development, characterized by poor soil structure, low organic matter content, weak microbial activity, and unstable nutrient cycling compared with long-term cultivated soils (Feng et al., 2024; Li et al., 2021a). These soils usually lack well-developed aggregates and mineral-associated carbon fractions, making them highly responsive to external organic inputs (Liu et al., 2025). During the initial years after reclamation, soil microbial communities and enzyme systems undergo rapid succession and restructuring, which strongly influence the formation of stable soil organic carbon pools (Li J. et al., 2025; Xiao et al., 2025). Investigating SOC stabilization processes in newly reclaimed farmland is therefore essential for understanding how management practices such as organic fertilization can accelerate soil quality restoration and carbon sequestration at the early stage of agricultural utilization. This context also provides an ideal system for disentangling the coupled microbial and physicochemical controls on carbon transformation and stabilization.

Although organic fertilizers have been widely shown to improve soil nutrients and promote SOC sequestration, the underlying mechanisms in newly reclaimed farmland remain unclear. The response patterns of different carbon fractions to organic inputs remain unclear, particularly regarding whether the conversion of active carbon to stable carbon is constrained by carbon saturation—a question for which evidence is still lacking. At the same time, the regulatory roles of microbial communities and key enzymes in this process remain poorly understood. Moreover, little is known about how organic fertilization affects SOC stabilization in such soils, where soil properties and microbial communities are still adjusting after reclamation. To address these knowledge gaps, we conducted a field experiment at a site that was reclaimed as arable land in 2023, providing a unique opportunity to investigate the early responses of soil carbon pools and microbial processes to organic inputs. Therefore, this study evaluated the effects of different organic fertilizer application rates on multiple SOC fractions. By integrating microbial and enzymatic analyses, we explored the coupling mechanisms between active carbon and stable carbon, providing new insights into carbon stabilization during the early stage of land reclamation and offering a basis for optimizing fertilizer management to balance productivity and environmental benefits.

## Materials and methods

### Experimental site

The field trial was conducted in Changhe Village (31°08′ N, 104°38′ E), Yongtai Township, Deyang City, Sichuan Province, China. The site had been left uncultivated until 2023, when cultivation began with the corn-growing season. The region falls within a subtropical humid climate zone, characterized by a mean annual temperature of 17.1 °C, average annual precipitation of 798.1 mm, and approximately 1,155 h of sunshine per year. The experimental field is dominated by purplish upland soil, classified as Eutric Cambisol according to the World Reference Base for Soil Resources. Prior to the experiment, baseline soil samples were collected to determine initial physicochemical properties. The soil was identified as loam, consisting of 31.5% sand, 58.8% silt, and 9.7% clay. Within the 0–20 cm plow layer, the soil had a pH of 7.5, bulk density of 1.25 g cmł, organic matter content of 8.5 g kg^−1^, total nitrogen of 0.92 g kg^−1^, available phosphorus of 54.0 mg kg^−1^, and available potassium of 186.0 mg kg^−1^.

### Experimental treatments and plot layout

The field experiment was conducted under a maize–oilseed rape rotation system, with soil samples for the oilseed rape season collected in May 2024 and May 2025. Each experimental plot had an area of 65 m^2^. The treatments comprised conventional synthetic fertilizer application and varying rates of organic fertilizer application: (1) CF: the recommended application rate of synthetic fertilizers for the study area (195 kg ha^–1^ of nitrogen, 97.5 kg ha^–1^ of P_2_O_5_, and 45 kg ha^–1^ of K_2_O for oilseed rape seasons, 162 kg ha^–1^ of nitrogen, 48 kg ha^–1^ of P_2_O_5_, and 30 kg ha^–1^ of K_2_O for maize seasons); (2) MF225: organic fertilizer applied at 3375 kg ha^–1^ (135 kg ha^–1^ of nitrogen, 101.3 kg ha^–1^ of P_2_O_5_, and 118.1 kg ha^–1^ of K_2_O for oilseed rape seasons, 96 kg ha^–1^ of nitrogen, 72 kg ha^–1^ of P_2_O_5_, and 84 kg ha^–1^ of K_2_O for maize seasons); 3) MF450: organic fertilizer applied at 6,750 kg ha^–1^ (270 kg ha^–1^ of nitrogen, 202.5 kg ha^–1^ of P_2_O_5_, and 236.3 kg ha^–1^ of K_2_O for oilseed rape seasons, 192 kg ha^–1^ of nitrogen, 144 kg ha^–1^ of P_2_O_5_, and 168 kg ha^–1^ of K_2_O for maize seasons); (4) MF900: organic fertilizer applied at 13,500 kg ha^–1^ (540 kg ha^–1^ of nitrogen, 405 kg ha^–1^ of P_2_O_5_, and 472.5 kg ha^–1^ of K_2_O for oilseed rape seasons, 384 kg ha^–1^ of nitrogen, 288 kg ha^–1^ of P_2_O_5_, and 336 kg ha^–1^ of K_2_O for maize seasons); (5) CF_MF225: synthetic fertilizers + 3,375 kg ha^–1^ organic fertilizer (330 kg ha^–1^ of nitrogen, 198.8 kg ha^–1^ of P_2_O_5_, and 163.1 kg ha^–1^ of K_2_O for oilseed rape seasons, 258 kg ha^–1^ of nitrogen, 120 kg ha^–1^ of P_2_O_5_, and 114 kg ha^–1^ of K_2_O for maize seasons). All treatments were arranged in triplicate in a randomized block design. Prior to transplanting, all fertilizers were applied as a single basal dose, and field management practices followed local recommendations for high-yield cultivation. The chemical fertilizer used was a rapeseed-specific compound fertilizer (Taiwo Co., China), while the organic fertilizer was produced from the fermentation of fungal residues and crop straw. Among them, the compound fertilizer contained 26%, 13%, and 6% of N, P2O5, and K2O for the oilseed rape season, and 27%, 8%, and 5% for the maize season, whereas the organic fertilizer contained 4%, 3%, and 3.5%, respectively, with a carbon content of 17.4%.

### Determination of soil carbon and corresponding enzymes

Oilseed rape was harvested by hand, and all grains from each plot were collected for yield determination. Fresh grain weight was recorded directly in the field. A representative subsample was subsequently oven-dried at 65 °C to a constant weight to measure moisture content. Plot yield was calculated by correcting the fresh weight with the measured moisture level and the standard reference moisture of 9% for oilseed rape. The dried subsamples were then finely ground with a ball mill, sieved through 0.5 mm mesh, and analyzed for carbon concentration using an elemental analyzer (Thermo Finnigan, Italy). After harvest, soil samples were collected from the 0–20 cm layer at random positions within each plot using a soil auger. Dissolved organic carbon (DOC) was extracted by leaching with distilled water, filtered through a 0.45 μm membrane, and quantified with an elemental analyzer according to Ghani et al. (2003). Readily oxidizable carbon (ROC) was determined using 333 mmol L^−1^ potassium permanganate as the oxidant and measured colorimetrically with a UV spectrophotometer following Blair et al. (1995). Particulate organic carbon (POC, ≥ 53 μm) and mineral-associated organic carbon (MAOC, < 53 μm) were separated following Cotrufo et al. (2019). Soil was dispersed in 5% sodium hexametaphosphate, shaken for 18 h, and then fractionated by wet sieving. The obtained fractions were oven-dried and their carbon concentrations determined using an elemental analyzer (Thermo Finnigan, Italy). Total soil organic carbon (SOC) was analyzed using the potassium dichromate-external heating method and quantified with a UV spectrophotometer (Thermo Fisher, United states) following Bao (2000).

Soil carbon sequestration (SCF) was calculated as the product of SOC concentration, bulk density, and soil depth at 0–20 cm (Bhatia et al., 2005). Plant carbon fixation (PCF) in oilseed rape was determined from grain yield and its carbon content (Bhatia et al., 2005). The total carbon fixation (TCF) of the soil–plant system was obtained by adding SCF and PCF. The efficiency of carbon fixation (TCFE) in the cropping system was expressed as the ratio of TCF to total carbon input (CI), which indicates how effectively applied carbon is converted into sequestration.

The following equations were used:

(a) SCF = SOC × BD × D

(b) PCF = Y_(grain)_ × C_(grain)_

(c) TCF = SCF + PCF

(d) TCFE = TCF/CI

where SOC is soil organic carbon concentration, BD is bulk density, D is sampling depth (20 cm), Y_(grain)_ is grain yield, C_(grain)_ is grain carbon content, and CI is total carbon input from fertilization.

Cellobiohydrolase (CBH) activity was assayed using p-nitrophenyl-β-D-cellobioside as the substrate (Saiya-Cork et al., 2002). Laccase (LAC) activity was determined with 2,2’-azino-bis (3-ethylbenzothiazoline-6-sulfonic acid) (ABTS) as the substrate (Eichlerová et al., 2012). Polyphenol oxidase (PPO) activity was measured using pyrogallol as the substrate (Bach et al., 2013).

### Soil DNA preparation and high-throughput sequencing

Total genomic DNA was extracted using the MagBeads FastDNA Kit for Soil (116564384) (MP Biomedicals, CA, United States) according to the manufacturer’s protocol and stored at −20 °C until analysis. DNA concentration and purity were determined with a NanoDrop NC2000 spectrophotometer (Thermo Fisher Scientific, Waltham, MA, United States), and integrity was verified by agarose gel electrophoresis.

Bacterial community diversity was analyzed by amplifying the V3–V4 hypervariable regions of the 16S rRNA gene using the forward primer 5′-ACTCCTACGGGAGGCAGCA-3′ and reverse primer 5′-GGACTACHVGGGTW TCTAAT-3′. Sample-specific 7-bp barcodes were incorporated into primers to enable multiplex sequencing. PCR amplification was carried out in a 25 μL reaction mixture containing 5 μL of 5 × Reaction Buffer, 5 μL of 5 × High GC Buffer, 0.25 μL of Q5 high-fidelity DNA polymerase (New England Biolabs), 2 μL of dNTPs (10 mM), 1 μL each of forward and reverse primers (10 μM), 2 μL of DNA template, and 8.75 μL of nuclease-free water. The thermal cycling program consisted of an initial denaturation at 98 °C for 5 min, followed by 25 cycles of denaturation at 98 °C for 30 s, annealing at 52 °C for 30 s, and extension at 72 °C for 45 s, and a final extension at 72 °C for 5 min. PCR products were purified with VAHTS DNA Clean Beads (Vazyme, Nanjing, China) and quantified using the Quant-iT PicoGreen dsDNA Assay Kit (Invitrogen, Carlsbad, CA, United States). Equimolar amounts of purified amplicons were then pooled and sequenced (2 × 300 bp paired-end) on an Illumina MiSeq platform (Illumina, United States) at Shanghai Personal Biotechnology Co., Ltd., (Shanghai, China) (Caporaso et al., 2012).

### Data analysis

Statistical analyses were conducted in R (version 4.5.1) to evaluate soil physicochemical properties. One-way analysis of variance (ANOVA) was performed to test for treatment effects, and significant differences among means were determined using Duncan’s multiple range test at *P* < 0.05. Prior to ANOVA, the Shapiro–Wilk test and Levene’s test were applied to verify normality and homogeneity of variances, ensuring that the assumptions of the analysis were satisfied. Graphical visualizations were produced using OriginPro 2025. In addition, relationships between soil carbon fractions were examined with the Mantel test implemented in the “linkET” package in R.

## Results

### Labile carbon fractions and soil organic carbon

**FIGURE 1:**
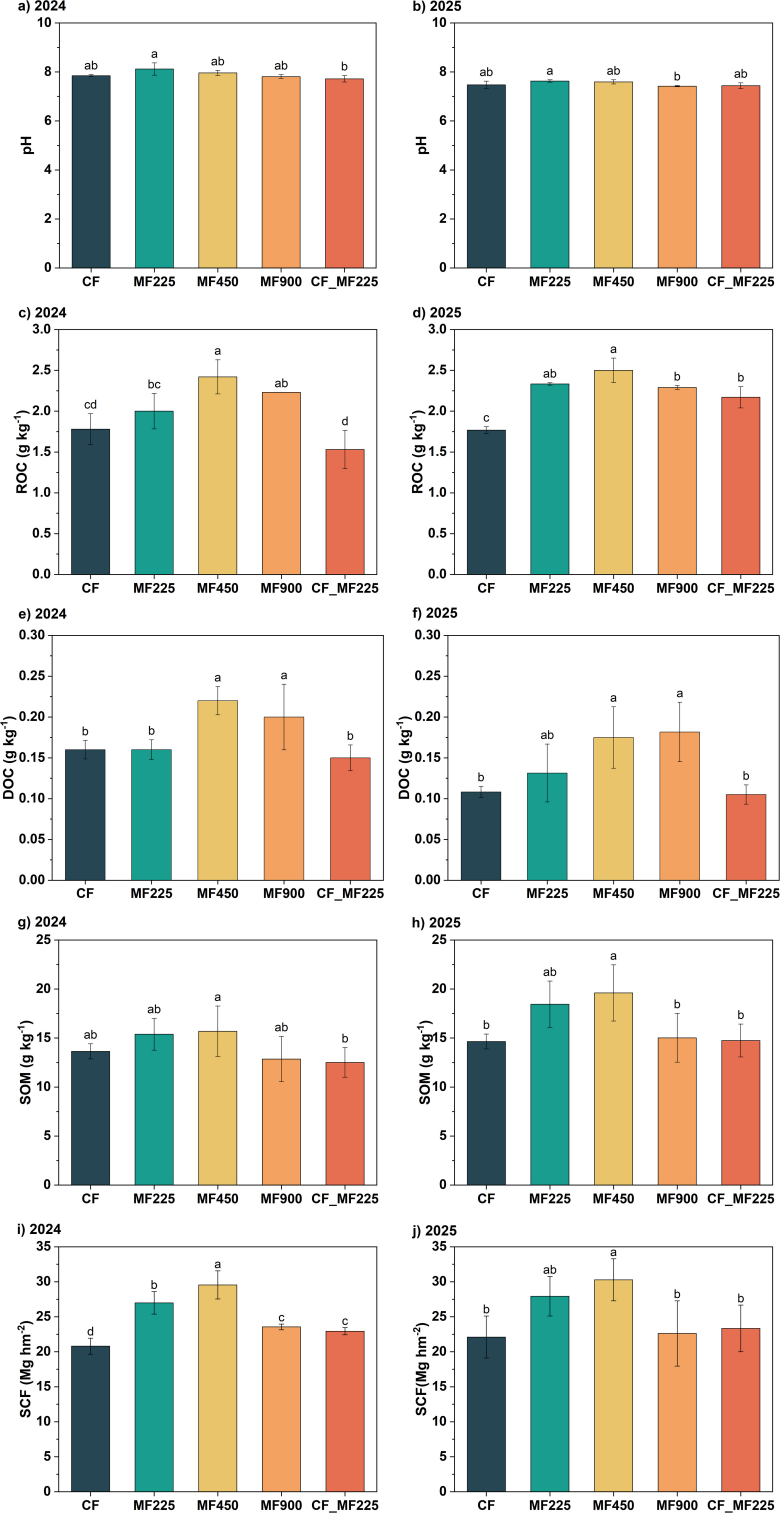
Effects of fertilization treatments on soil pH **(a,b)**, readily oxidizable organic carbon [ROC, **(c,d)**], dissolved organic carbon [DOC, **(e,f)**], soil organic matter [SOM, **(g,h)**], and soil carbon sequestration [SCF, **(i,j)**] in 2024 and 2025. Different letters indicate significantly different values (*P* < 0.05).

To assess the effects of organic fertilizer application on soil physicochemical properties and carbon sequestration, we measured soil pH and carbon fractions during the 2024 and 2025 oilseed rape seasons and calculated soil carbon sequestration. Compared with CF (Figures 1a, b), organic fertilizer application slightly increased soil pH in both years, although the differences were not statistically significant. For ROC, in 2024 (Figure 1c), only MF450 and MF900 significantly increased its content by 36.0% and 25.3%, respectively, while the other treatments showed no significant differences. In 2025 (Figure 1d), however, all organic fertilizer treatments significantly increased ROC, with increases of 32.0%, 41.4%, 29.5%, and 22.7% for MF225, MF450, MF900, and CF+MF225, respectively. Notably, DOC exhibited a similar trend in both years. Specifically, MF450 and MF900 significantly increased DOC by 37.5% and 25.0% in 2024 (Figure 1e), and by 61.7% and 67.9% in 2025 (Figure 1f), respectively, whereas the other treatments did not differ significantly from CF in either year. In contrast, organic fertilizer application had no significant effect on soil organic matter (SOM) in 2024 (Figure 1g), whereas in 2025 (Figure 1h), only MF450 significantly increased SOM by 33.8%. To further evaluate the enhancement of soil carbon sequestration, we calculated the soil carbon sequestration (SCF). In 2024 (Figure 1i), all organic fertilizer treatments significantly increased SCF by 29.8%, 42.1%, 13.2%, and 10.3% for MF225, MF450, MF900, and CF_MF225, respectively. However, in 2025 (Figure 1j), only MF450 significantly increased SCF by 37.0%, while the other treatments had no significant effects.

### Changes in soil organic carbon fractions

**FIGURE 2:**
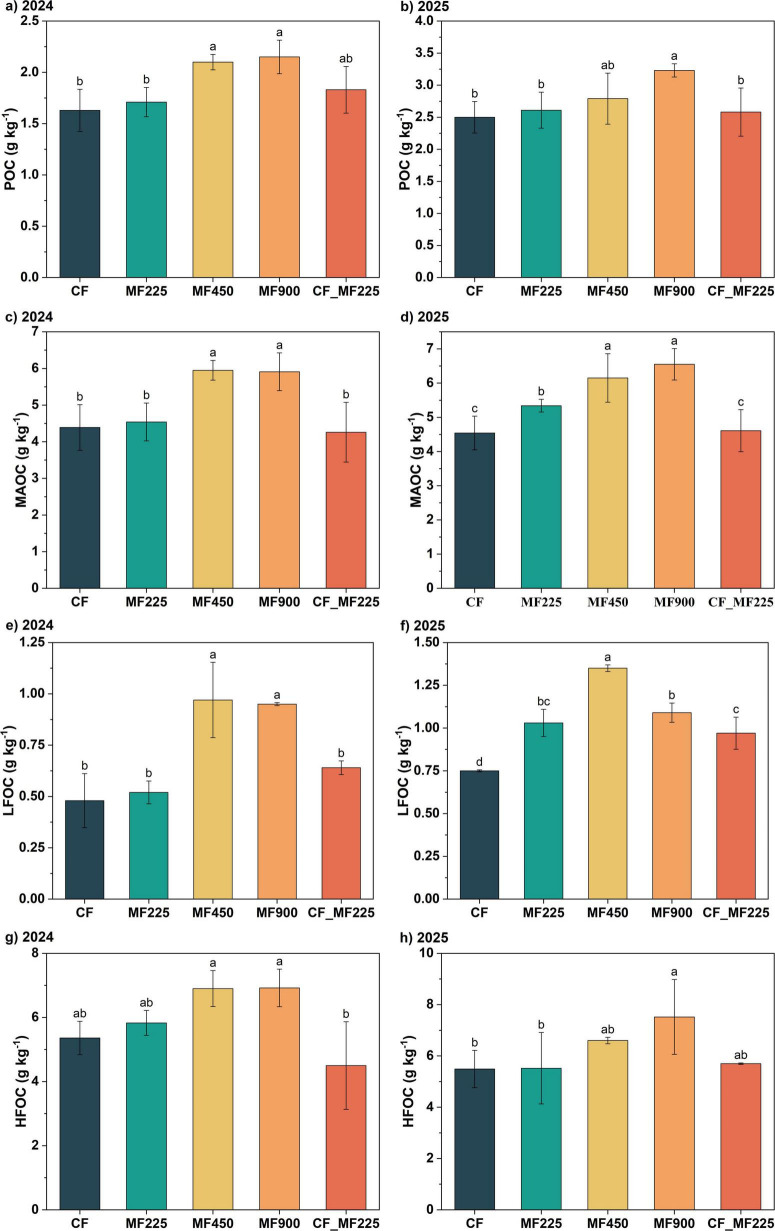
Effects of fertilization treatments on particulate organic carbon [POC, **(a,b)**], mineral-associated organic carbon [MAOC, **(c,d)**], light-fraction organic carbon [LFOC, **(e,f)**], and heavy-fraction organic carbon [HFOC, **(g,h)**] in 2024 and 2025. Different letters indicate significantly different values (*P* < 0.05).

To assess the impact of organic fertilizer application on soil carbon stabilization, we analyzed soil organic carbon fractions. Organic fertilizer application increased POC, MAOC, LFOC, and HFOC contents in both 2024 and 2025 compared with CF, with the most pronounced effects observed under higher application rates. Notably, only MF900 significantly increased POC in both years, by 31.9% and 29.2% (Figures 2a, b), while MF450 significantly increased POC by 28.8% in 2024 and by 11.6% in 2025, though the latter was not statistically significant. The other organic fertilizer treatments did not result in significant differences in POC compared with CF. For MAOC (Figures 2c, d), only MF450 and MF900 significantly increased its content in 2024, by 35.5% and 34.6%, respectively, whereas in 2025, MF225, MF450, and MF900 significantly increased MAOC by 17.6%, 35.5%, and 44.3%, respectively. In contrast, CF_MF225 did not lead to significant changes in either year. LFOC exhibited a similar pattern to MAOC in 2024 (Figures 2e,f), with only MF450 and MF900 significantly increasing its content by 35.5% and 34.6%, respectively, while the other treatments showed no significant differences. However, in 2025, all organic fertilizer treatments significantly increased LFOC, with MF225, MF450, MF900, and CF_MF225 increasing its content by 37.3%, 80.0%, 45.3%, and 29.3%, respectively. HFOC followed a trend similar to LFOC in 2024 (Figures 2g, h), with only MF450 and MF900 significantly increasing its content by 28.7% and 29.1%, respectively, whereas in 2025, only MF900 achieved a significant increase.

### Crop yield and carbon fixation

**FIGURE 3:**
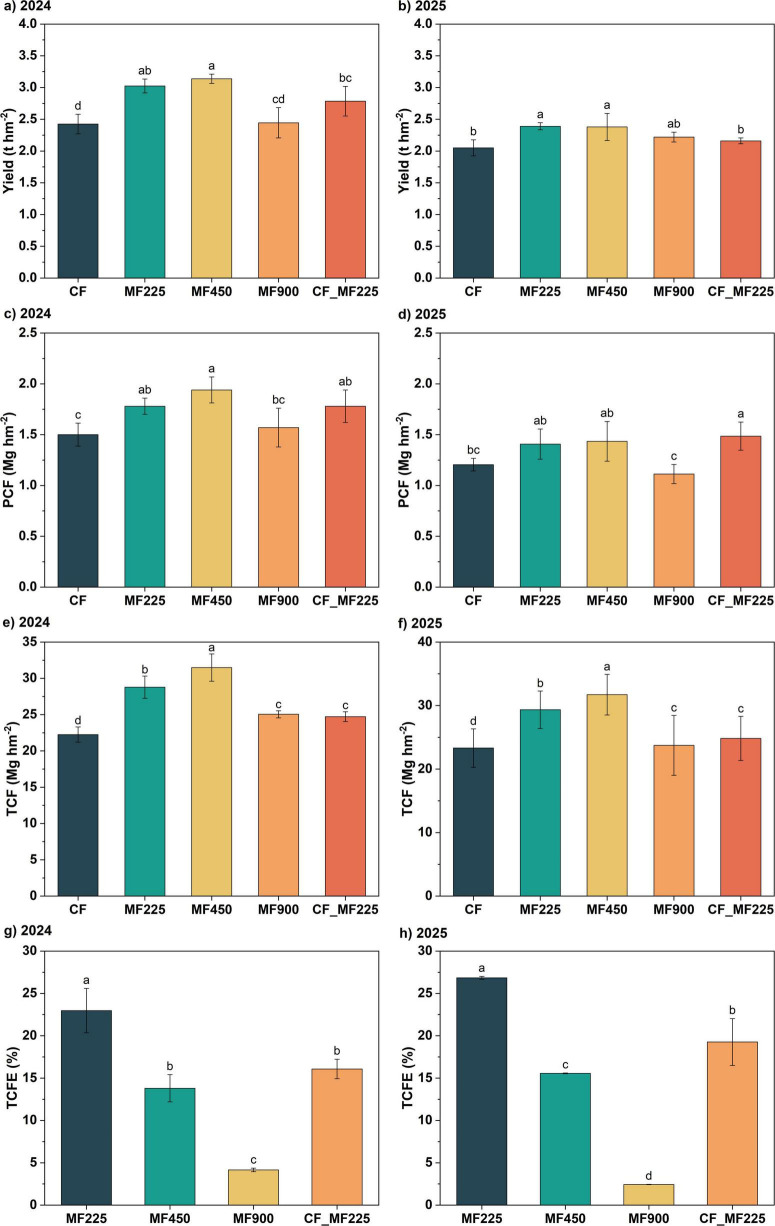
Effects of fertilization treatments on yield **(a,b)**, plant carbon fixation [PCF, **(c,d)]**, total carbon fixation [TCF, **(e,f)]**, and total carbon fixation efficiency [TCFE, **(g,h)]** in 2024 and 2025. Different letters indicate significantly different values (*P* < 0.05).

Through the 2-year data on oilseed rape yield, we found that, compared with CF, organic fertilizer application increased yield to varying degrees, with a more pronounced effect in 2024, while the yield improvement declined in 2025. Specifically, in 2024 (Figure 3a), all fertilizer treatments increased yield relative to CF, with MF225, MF450, and CF_MF225 significantly increasing yield by 24.7%, 29.4%, and 14.9%, respectively, while MF900 did not differ significantly from CF. In 2025 (Figure 3b), however, only MF225 and MF450 significantly increased yield by 16.6% and 16.1%, respectively, whereas the other organic fertilizer treatments increased yield without reaching statistical significance. As shown in Figures 3c, d, SCF exhibited a consistent pattern across both years, with organic fertilizer application enhancing PCF relative to CF. Among the treatments, MF450 achieved the highest PCF, whereas MF900 did not produce a significant improvement compared with CF. Specifically, MF225, MF450, and CF_MF225 increased PCF by 18.7%, 29.3%, and 18.7% in 2024, and by 16.8%, 19.0%, and 23.3% in 2025, respectively. Soil-plant total carbon sequestration (TCF) exhibited a trend similar to that of plant carbon sequestration (PCF) (Figures 3e, f), with organic fertilizer application significantly increasing TCF in both years. Among the treatments, MF900 achieved the highest TCF, followed by MF450. In contrast, calculation of total carbon sequestration efficiency (TCFE) showed that MF225 consistently exhibited the highest TCFE across both years, followed by CF_MF225, while MF900 had the lowest TCFE (Figures 3g, h).

### Enzyme activity

**FIGURE 4:**
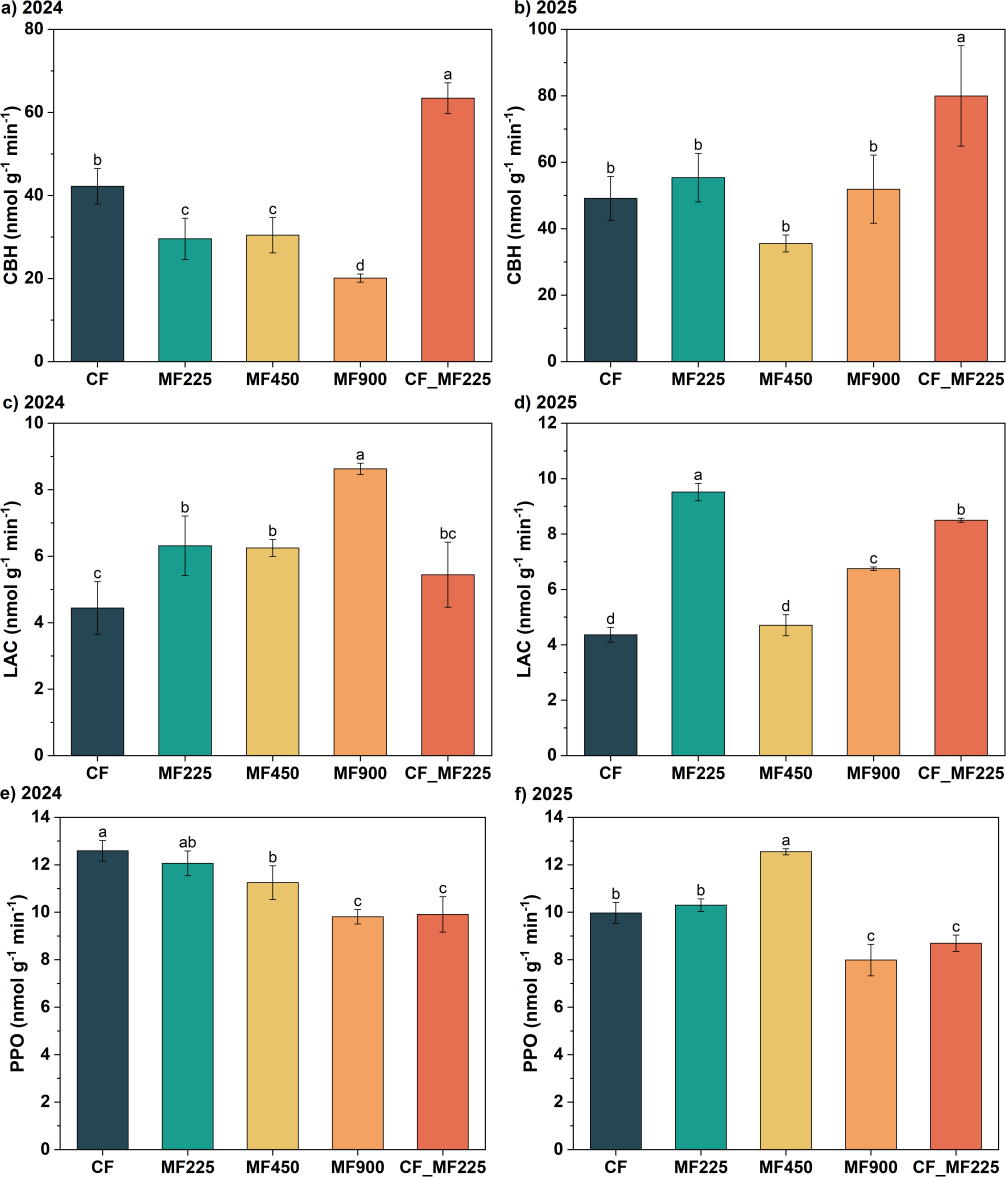
Effects of fertilization treatments on cellobiohydrolase [CBH, **(a,b)]**, laccase [LAC, **(c,d)]**, and polyphenol oxidase [PPO, **(e,f)]** activities in 2024 and 2025. Different letters indicate significantly different values (*P* < 0.05).

To evaluate changes in soil enzyme activities associated with carbon cycling, we measured cellobiohydrolase (CBH), laccase (LAC), and polyphenol oxidase (PPO) over the 2-year period. The three enzymes exhibited distinct temporal trends. Specifically, in 2024 (Figure 4a), compared with CF, sole organic fertilizer applications (MF225, MF450, and MF900) decreased CBH activity by 30.0%, 27.9%, and 52.4%, respectively, whereas the combined chemical–organic fertilizer treatment (CF_MF225) increased CBH activity by 50.2%. In 2025 (Figure 4b), although CBH activity under MF225 increased compared with 2024, it did not differ significantly from CF, while CF_MF225 continued to enhance CBH activity by 44.4%. For LAC (Figures 4c, d), organic fertilizer treatments increased enzyme activity in both years, with MF900 achieving the highest increase of 94.3% in 2024 and MF225 showing the highest increase of 118.0% in 2025. In contrast, PPO activity decreased under organic fertilizer treatments compared with CF in 2024 (Figure 4e), whereas in 2025 (Figure 4f), MF450 exhibited a higher PPO activity than CF.

### Soil microbial community

**FIGURE 5:**
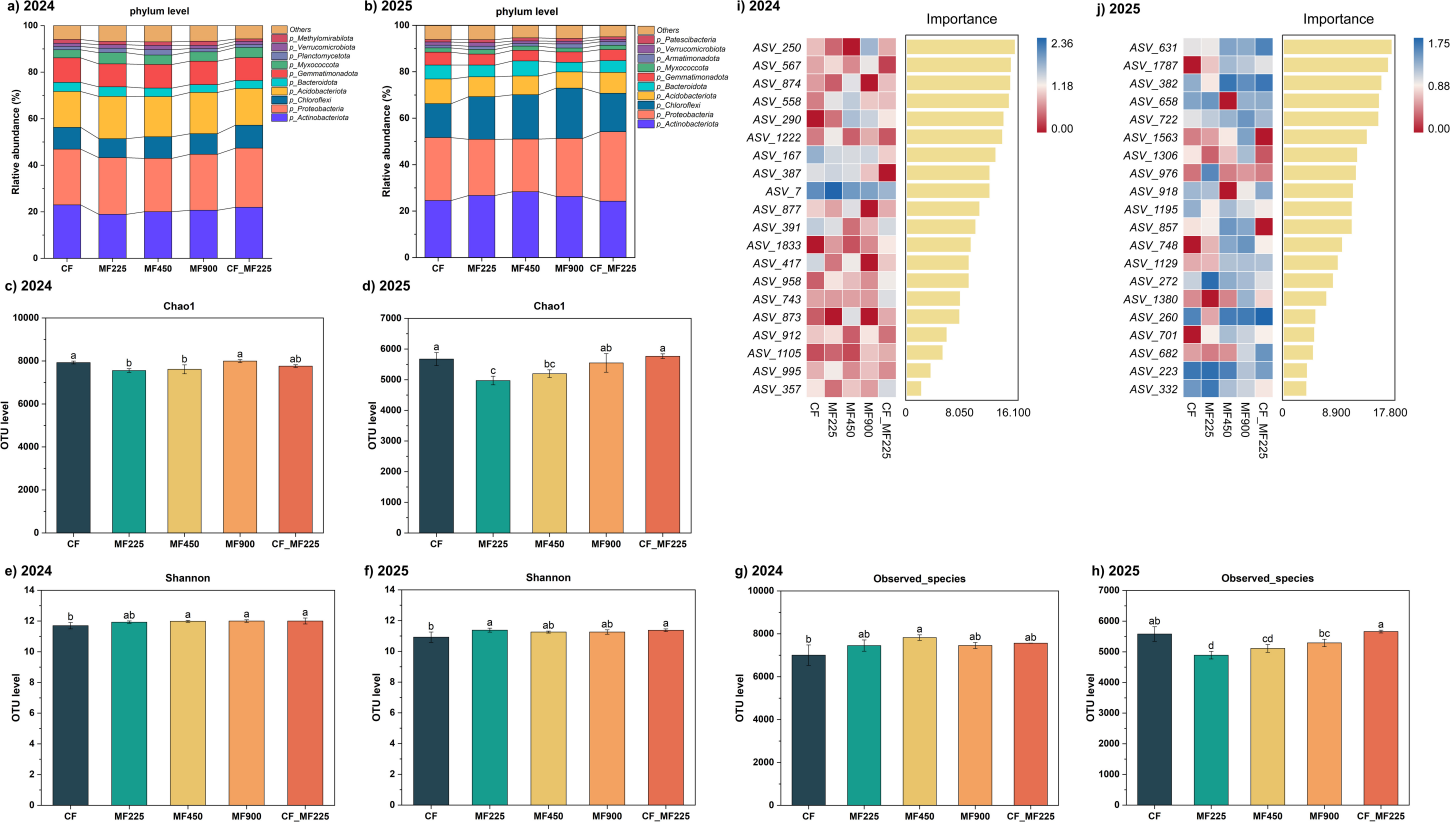
Effects of fertilization treatments on microbial community and diversity indices in 2024 and 2025. Microbial community structure and species composition **(a,b)**, Chao1 index **(c,d)**, Shannon index **(e,f)**, observed species richness **(g,h)**, and random forest analysis of top discriminant ASVs **(i,j)**, showing heatmap-based relative abundances and random forest importance scores, with the color scale indicating standardized abundance: blue = high, red = low). Different letters indicate significantly different values (*P* < 0.05).

At the phylum level, *Actinobacteriota* and *Proteobacteria* were the dominant groups across all treatments in both years (Figures 5a, b). In 2024, organic and combined fertilization reduced the relative abundance of *Actinobacteriota* compared with CF, whereas in 2025 its abundance increased under fertilized treatments. In contrast, *Proteobacteria* was consistently higher than CF in both years, with the largest increases observed under MF225, MF900, and CF_MF225. Thus, relative to CF, organic and combined fertilization shifted the community toward higher *Proteobacteria*, while the response of *Actinobacteriota* was year-dependent. Other phyla such as *Chloroflexi* and *Acidobacteriota* showed slight increases in 2025, while *Bacteroidota* declined, and low-abundance groups (*Gemmatimonadota*, *Myxococcota*, *Verrucomicrobiota*) remained stable at < 5%.

In line with the shifts in community composition, α diversity indices also varied with fertilization treatments (Figures 5c–h). For the Chao1 index (Figures 5c, d), values under MF225 and MF450 were consistently lower than CF in both 2024 and 2025, while MF900 and CF_MF225 remained comparable to CF. The reduction was more evident in 2025, with MF225 showing a marked decline and MF450 a moderate decrease. For the Shannon index (Figures 5e, f), diversity under organic and combined fertilization was generally higher than under CF. In 2024, MF450, MF900, and CF_MF225 showed significantly higher Shannon values than CF, whereas MF225 was comparable. In 2025, the pattern shifted, with MF225 and CF_MF225 reaching significant increases over CF. For observed species richness (Figures 5g, h), organic and combined fertilization were overall higher than CF in 2024, but only MF450 reached a significant increase of 11.7%. In 2025, however, the trend reversed: MF225 and MF450 were significantly lower than CF by 12.3% and 8.4%, respectively, while MF900 and CF_MF225 followed the same pattern as in 2024.

Random forest analysis identified distinct sets of discriminant ASVs in 2024 and 2025 (Figures 5i, j). In 2024, the most important taxa included *ASV_250*, *ASV_567*, *ASV_874*, and *ASV_558*, all of which had high importance scores (> 12) and were enriched under organic fertilization compared with CF. In 2025, however, a different group of ASVs dominated the importance ranking, such as *ASV_631*, *ASV_1787*, and *ASV_382*, with importance scores approaching or exceeding those observed in 2024. These ASVs showed variable abundance patterns, with some enriched under CF while others increased under organic treatments. Despite the shift in specific ASVs between years, the high importance values across both years indicate that organic and combined fertilization consistently selected for key microbial taxa that distinguished them from CF.

### Relationship between soil carbon fractions and microbial communities

**FIGURE 6:**
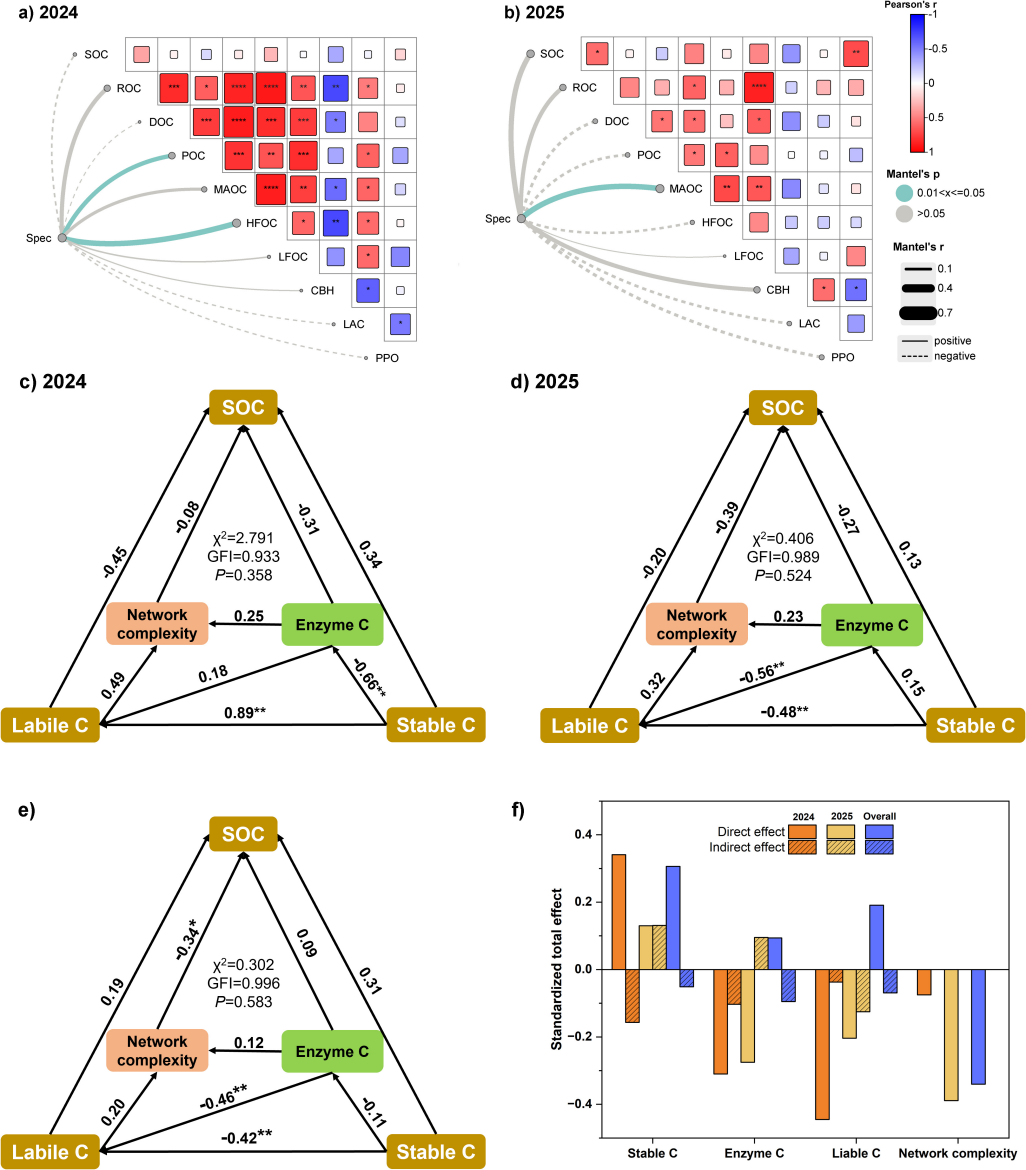
Relationships between soil properties, microbial diversity, and soil organic carbon (SOC) accumulation as revealed by Mantel test and structural equation modeling (SEM) in 2024, 2025, and their overall combination. **(a,b)** Show Mantel test results linking bacterial community composition at the genus level (Spec) with soil physicochemical indicators in 2024 and 2025, respectively. **(c–e)** Present SEM pathways for SOC accumulation in 2024, 2025, and the overall dataset, with numbers on arrows indicating standardized path coefficients. *, **, ***, and **** denote significance at *p* < 0.05, *p* < 0.01, *p* < 0.001, and *p* < 0.0001, respectively. Labile C and Stable C represent labile and stable carbon fractions, Enzyme C indicates carbon-cycle-related enzyme activities, and Network complexity denotes microbial co-occurrence network complexity. GFI refers to the goodness-of-fit index. **(f)** Summarizes the standardized total effects (STE) of different factors on SOC, partitioned into direct and indirect effects for 2024, 2025, and the overall combination.

Heatmap analysis revealed distinct correlation patterns between SOC fractions and enzyme activities in 2024 (Figure 6a) and 2025 (Figure 6b). During 2024, POC, MAOC, and HFOC were strongly and positively correlated with total SOC, DOC, and ROC, while POC and MAOC also showed a significant positive association with each other, underscoring the linkage between labile and stable carbon pools. In contrast, CBH and LAC displayed only weak correlations with SOC fractions, whereas PPO was negatively related to several carbon pools. In 2025, MAOC continued to be positively associated with SOC and other labile fractions, but the three enzymes (CBH, LAC, PPO) showed only limited relationships with carbon pools. Mantel tests indicated that microbial community composition at the genus level (Spec) was significantly correlated with POC and HFOC in 2024, whereas in 2025 the correlation was restricted to MAOC.

Structural equation modeling (SEM) offered further insight into the mechanisms underlying SOC accumulation (Figures 6c–f). In 2024 (Figure 6c), labile C exerted a strong positive direct effect on stable C (path coefficient = 0.89, *P* < 0.01), while enzyme C was negatively associated with stable C (−0.66, *P* < 0.01). Network complexity contributed only marginally. In 2025 (Figure 6d), the regulatory pattern shifted, with labile C exerting strong negative effects on both enzyme C (−0.56, *P* < 0.01) and stable C (−0.48, *P* < 0.01), suggesting that excessive labile inputs suppressed stabilization.

At the integrated level (Figure 6e), results were consistent with the year-specific models. Labile C negatively influenced stable C (−0.42, *P* < 0.01) and was itself suppressed by network complexity (−0.46, *P* < 0.05), highlighting the role of microbial interactions in carbon turnover.

The standardized total effect (STE) analysis (Figure 6f) clarified these dynamics further. In 2024, stable C contributed positively to SOC accumulation, while enzyme C acted as a negative regulator. In 2025, labile C and network complexity emerged as the main inhibitory factors, and the influence of stable C and enzyme C diminished. Across models, network complexity consistently imposed negative effects, suggesting that highly complex microbial networks may accelerate carbon cycling rather than enhance long-term stabilization.

## Discussion

The application of organic fertilizer markedly modified soil carbon dynamics, exerting significant impacts on both labile and stable carbon pools. In this study, organic fertilizer treatments increased ROC and DOC in both years, with the strongest responses observed under medium (MF450) and high (MF900) application rates. These results demonstrate that organic inputs supply readily available substrates that directly enhance soil labile carbon pools. Nevertheless, the long-term stability of soil organic matter (SOM) depends not simply on the accumulation of labile fractions but on their conversion into stable pools such as MAOC and HFOC. Our findings revealed that MF450 and MF900 consistently promoted the formation of MAOC and HFOC, whereas excessive labile inputs, particularly in 2025, inhibited stable carbon formation. Structural equation modeling (SEM) further verified these patterns. In 2024, labile carbon exerted a positive effect on stable carbon, but in 2025 the relationship became negative. This shift suggests that carbon stabilization relies on maintaining a balance between substrate supply and microbial processing capacity. The time-dependent effects show the dual role of labile carbon. It serves as a precursor that supports stabilization under moderate inputs, but it promotes rapid mineralization when inputs are excessive (Chivenge et al., 2011; Kuzyakov, 2010). Such reversals in regulatory pathways over time are rarely reported in previous studies. They provide new evidence that the effects of organic inputs on SOC are constrained by time and by input level (Gattinger et al., 2012; Holatko et al., 2022).

Changes in soil enzyme activities give further support to this interpretation CBH activity went down under treatments with only organic fertilizer, showing less cellulose degradation and a lower release of labile carbon. But when chemical and organic fertilizers were applied together (CF_MF225), CBH activity went up, pointing to a synergistic effect that helps keep the turnover of plant residues more balanced. LAC, an oxidative enzyme linked to lignin and aromatic compound breakdown, showed clear increases under organic fertilization, most strongly with MF900 in 2024 and MF225 in 2025. This shows that organic inputs push microbes to degrade recalcitrant carbon, which may favor its change into mineral-associated forms. PPO showed mixed results. Its activity went down with organic treatments in 2024 but went up with MF450 in 2025. This likely reflects shifts in microbial groups with different oxidative abilities (Burns et al., 2013; Trivedi et al., 2016). Together, these results show that organic fertilization changes not only the amount of carbon entering the soil but also the enzymes that control its use, and in this way, it reshapes the routes of carbon stabilization.

The changes in microbial communities provide deeper insight into the underlying mechanisms. Organic fertilization consistently increased the abundance of Proteobacteria, a group of fast-growing copiotrophs that rely on easily degradable carbon sources (Fierer et al., 2007; Guo et al., 2025). Organic inputs favor microbes that use readily available nutrients, and this accelerates short-term carbon cycling. But when these fast-growing microbes dominate, they may suppress slower-growing groups and shift the balance between rapid carbon breakdown and long-term carbon stabilization in soils (Fierer et al., 2007; Nemergut et al., 2010). *Actinobacteriota* showed clear interannual variation: their abundance declined in 2024 but increased in 2025. This suggests that their role in decomposition is more closely linked to long-term nutrient supply and the gradual buildup of crop residues (Meng et al., 2025). Other phyla, such as *Chloroflexi* and *Acidobacteriota*, showed modest increases in 2025, which indicates possible roles in decomposition under shifting resource conditions (Zhang X. et al., 2024). Microbial diversity also changed. Shannon diversity rose under organic fertilization, while species richness (Chao1) declined at low input levels. This suggests that organic amendments may enhance functional diversity rather than simply increase species numbers, likely by favoring specialized decomposers able to process complex organic materials (Francioli et al., 2016; Whalen et al., 2024). Network analyses further show that fertilization reshapes microbial communities. It strengthens certain functional groups but at the same time reduces overall balance among species (Li et al., 2023; Raimi et al., 2023).

Network and SEM analyses provide a combined view of the interactions among carbon fractions, enzymes, and microbial communities. In 2024, positive links between labile fractions (POC, LFOC) and stable carbon pools suggested that microbes played a key role in driving efficient stabilization processes. In contrast, in 2025, higher network complexity combined with excessive inputs of easily degradable carbon actually reduced SOC accumulation. This finding challenges the common assumption that greater microbial diversity and more complex networks always lead to stronger carbon sequestration (Peng et al., 2024). Instead, they suggest that overly complex microbial interactions may accelerate carbon turnover and loss when substrate inputs surpass the system’s stabilization capacity. This mechanism is consistent with the “microbial carbon pump” framework, in which microbial activity governs whether carbon is stabilized or rapidly respired (Jiao et al., 2010; Liang et al., 2017). Similar evidence has recently emerged from soil network studies, showing that highly connected microbial networks can enhance nutrient turnover but also increase vulnerability of carbon pools under disturbance (Byers et al., 2025).

In terms of agronomic performance, organic fertilization markedly increased oilseed rape yield in 2024; however, this effect declined in 2025, particularly under high-input treatments. Among the treatments, MF225 consistently achieved the highest carbon sequestration efficiency (TCFE), indicating that moderate application rates best balance crop productivity and soil carbon storage. In contrast, excessive organic inputs (MF900) increased total carbon sequestration (TCF) but exhibited lower efficiency in carbon utilization, reflecting diminishing returns under high-input regimes (Xu et al., 2025). These results highlight the necessity of optimizing input levels and support recent evidence that balanced organic–inorganic fertilization improves carbon efficiency more effectively than high organic inputs alone (Li X. et al., 2025; Zhang et al., 2022).

## Conclusion

Our results demonstrate that organic fertilization enhanced SOC accumulation through substrate input, enzyme stimulation, and microbial regulation, but the effects were strongly dependent on application rate and year. Moderate inputs promoted stable carbon formation, whereas excessive additions reduced stabilization efficiency and increased microbial network complexity that negatively influenced long-term SOC storage. Importantly, because the field was reclaimed in 2023, the findings highlight that newly reclaimed soils are highly sensitive to organic fertilizer application rates, making input management especially critical during the early reclamation stage. Overall, shifts in microbial communities and their network interactions played a central role in these processes, with higher diversity and functional specialization supporting stabilization under moderate inputs, while excessive inputs and overly complex networks suppressed it. These insights provide a refined understanding of SOC stabilization mechanisms and practical guidance for managing organic inputs in reclaimed farmland to enhance both soil fertility and carbon sequestration.

## Data Availability

The datasets presented in this study can be found in online repositories. The names of the repository/repositories and accession number(s) can be found below: https://ngdc.cncb.ac.cn/gsa, CRA029942.
